# Invertebrate Trehalose-6-Phosphate Synthase Gene: Genetic Architecture, Biochemistry, Physiological Function, and Potential Applications

**DOI:** 10.3389/fphys.2018.00030

**Published:** 2018-01-31

**Authors:** Bin Tang, Su Wang, Shi-Gui Wang, Hui-Juan Wang, Jia-Yong Zhang, Shuai-Ying Cui

**Affiliations:** ^1^College of Life and Environmental Sciences, Hangzhou Normal University, Hangzhou, China; ^2^Department of Medicine, Boston University School of Medicine, Boston, MA, United States; ^3^Beijing Key Laboratory of Environment Friendly Management on Fruit Diseases and Pests in North China, Institute of Plant and Environment Protection, Beijing Academy of Agriculture and Forestry Sciences, Beijing, China; ^4^Key Lab of Wildlife Biotechnology, Conservation and Utilization of Zhejiang Province, College of Life Science and Chemistry, Zhejiang Normal University, Jinhua, China

**Keywords:** trehalose, trehalose-6-phosphate synthase, physiological function, chitin regulation, TPS inhibitor

## Abstract

The non-reducing disaccharide trehalose is widely distributed among various organisms. It plays a crucial role as an instant source of energy, being the major blood sugar in insects. In addition, it helps countering abiotic stresses. Trehalose synthesis in insects and other invertebrates is thought to occur via the trehalose-6-phosphate synthase (TPS) and trehalose-6-phosphate phosphatase (TPP) pathways. In many insects, the *TPP* gene has not been identified, whereas multiple *TPS* genes that encode proteins harboring TPS/OtsA and TPP/OtsB conserved domains have been found and cloned in the same species. The function of the *TPS* gene in insects and other invertebrates has not been reviewed in depth, and the available information is quite fragmented. The present review discusses the current understanding of the trehalose synthesis pathway, TPS genetic architecture, biochemistry, physiological function, and potential sensitivity to insecticides. We note the variability in the number of *TPS* genes in different invertebrate species, consider whether trehalose synthesis may rely only on the *TPS* gene, and discuss the results of *in vitro TPS* overexpression experiment. Tissue expression profile and developmental characteristics of the *TPS* gene indicate that it is important in energy production, growth and development, metamorphosis, stress recovery, chitin synthesis, insect flight, and other biological processes. We highlight the molecular and biochemical properties of insect TPS that make it a suitable target of potential pest control inhibitors. The application of trehalose synthesis inhibitors is a promising direction in insect pest control because vertebrates do not synthesize trehalose; therefore, TPS inhibitors would be relatively safe for humans and higher animals, making them ideal insecticidal agents without off-target effects.

## Trehalose and its function in invertebrates

Trehalose is a non-reducing disaccharide in which two glycosyl moieties are linked together by an α,α-1,1 bond (Elbein et al., 2003; Bansal et al., 2013). It is found ubiquitously as a metabolite in various bacteria, fungi, slime molds, protozoa, plants, and invertebrates (Kern et al., 2012; Tang et al., 2012a,b, 2014a,b, 2016; Lyu et al., 2013). Trehalose functions not only as a reserve carbohydrate, but also as an important stress-protecting molecule in different organisms (Elbein et al., 2003; Pampurova et al., 2014). Trehalose has been shown to serve as a mobile energy source for flight, and its levels in the blood have been reported to control the expenditure of flight energy in insects (Clegg and Evans, 1961; Cui and Xia, 2009). High levels of trehalose are also present in the hemolymph of insects at nonflying stages and in the blood of invertebrates that use lipids for flight energy (Wyatt, 1961). The levels of blood trehalose vary greatly in the developmental history of different species, and in all probability, trehalose has been adapted for diverse functions within the class Insecta (Murphy and Wyatt, 1965). However, trehalose synthesis pathway has not been found in higher animals (mammals) or vertebrates, even though trehalase (TRE) has been reported in the small intestine, digestive system, and other organs of various species, especially in insects and other invertebrates (Richards et al., 2002; Chen and Haddad, 2004).

In the animal kingdom, trehalose was first identified as an important constituent of insect hemolymph in silkworm pupae (Wyatt and Kalf, 1956). Trehalose was then found in concentrations of up to 2% in the hemolymph of the desert locust *Schistocerca gregaria* (Howden and Kilby, 1956). This sugar is an important soluble carbohydrate and energy reserve in insects (Kandy and Kilby, 1959). It is secrected into the hemolymph of insects at all developmental stages (Matsuda et al., 2015). Trehalose functions as a source of glucose for energy in adult insects during flight and energy-requiring activities; it also serves as an energy source to meet the demands of insect flight muscles and other tissues and is continuously synthesized in the fat body (Evans and Dethier, 1957; Wyatt and Kalf, 1957; Bücher and Klingenberg, 1958; Candy and Kilby, 1961; Becker et al., 1996; Elbein et al., 2003; Chen and Haddad, 2004; Kern et al., 2012; Gao et al., 2014; Shukla et al., 2015). Trehalose serves not only as a reserve carbohydrate but also as an efficient protection factor, playing important roles in the protection of organisms against adverse environmental conditions (Iordachescu and Imai, 2008; Tang et al., 2008; Shukla et al., 2015; Liu et al., 2016). Trehalose is also essential for stress response in various microorganisms, and its inhibition may be a promising antimicrobial strategy as *TPS* genes are entirely absent in humans (Magalhães et al., 2017). Survival strategies for overwintering insects are determined by biochemical components of their body fluids. Freeze-tolerant and freeze-avoiding insects often accumulate a high level of trehalose that acts as a supercooling agent and cryoprotectant (Storey and Storey, 2012; Wen et al., 2016). During menadione stress, trehalose has been found to be necessary for yeast intracellular functions (Herdeiro et al., 2006), whereas the presence of trehalose on both sides of the lipid bilayer minimized oxidative damage to proteins and lipids (da Costa Morato Nery et al., 2008).

In nematodes, trehalose is usually present at a concentration higher than that of free glucose (Fairbairn, 1958; Dmitryjuk et al., 2009), and has many important functions: it protects cellular structures during stresses such as high osmotic pressure, drying, or freezing; it provides energy as the major circulating sugar; and it is important for egg hatching (Perry, 1989; Behm, 1997; Dmitryjuk and Zółtowska, 2003; Elbein et al., 2003). Nearly all insects maintain high level of trehalose in their hemolymph (Wyatt, 1967; Kramer et al., 1978; Becker et al., 1996; Mariano et al., 2009). Trehalose protects organisms against different environmental stresses, including heat, oxidation, cold, anoxia, or desiccation, because of its unique chemical properties (Crowe et al., 1998; Elbein et al., 2003; Matsuda et al., 2015). In *Drosophila* larvae desiccated for 10 h at <5% relative humidity, the desiccation-responsive trehalose metabolic pathway was activated in concert with the enzymes TPS and TRE (Shukla et al., 2015). These data indicate that trehalose is a potential marker for anhydrobiosis in *Drosophila* (Thorat et al., 2012).

As in mammals, insulin-like peptides (Dilps) and a glucagon-like peptide regulate circulating sugar levels in *Drosophila* (Yasugi et al., 2017). Feeding on dietary sugar immediately changes the levels of the circulating sugar (Ugrankar et al., 2015). Genetic manipulation of the function of Dilps and adipokinetic hormone (Akh) changes trehalose and glucose levels in the circulating hemolymph, which means that mobilization of blood trehalose to glucose is critical for metabolic homeostasis (Rulifson et al., 2002; Gáliková et al., 2015). Flight, feeding, and parasitic infections in insects produce hypertrehalosemia in the hemolymph (Becker et al., 1996; Zółtowska and Lopieniska-Biernat, 2006). These findings further support the notion that trehalose plays a role in the response to several biological functions as a physiological adaptations and as an energy source in insects (Chung, 2008).

In recent years, several approaches have been applied to study the trehalose synthesis genes *TPS* and *TPP*. Their special functions in molecular mechanisms underlying different stresses and even in the regulation of chitin synthesis have been described in insects and other invertebrates (Chen et al., 2002, 2003; Tang et al., 2010; Chen and Zhang, 2015; Shi et al., 2016; Xiong et al., 2016; Yang et al., 2017), taking advantage of their ability to adapt to stress conditions (Chen and Haddad, 2004; Qin et al., 2012; Tang et al., 2014b; Guo et al., 2015). On the one hand, an increasing number of *TPS* genes are being identified and cloned from different insects and other invertebrate species. On the other hand, many insects seem to possess only *TPS* genes but no *TPP* gene according to genome sequencing results. In this regard, several following questions arise. How does trehalose synthesis proceed in invertebrates and is there another pathway in addition to the TPS/TPP pathway? Is the presence of just the *TPS* gene sufficient for trehalose synthesis because the encoded protein has both TPS and TPP domains? Do trehalose synthesis pathways vary between different insects? In this review, we summarize and discuss the current knowledge of the invertebrate trehalose synthesis pathway; the cloning and expression of the underlying genes identified so far; their role in development, stress conditions, and chitin metabolism regulation. We also point out the knowledge gaps that need to be filled, especially regarding future pest control by using inhibitors of trehalose synthesis, considering the absence of TPS in vertebrates.

## Genetic architecture

### *TPS* and *TPP* gene identification, cloning, and evolution

**Figure 1:**
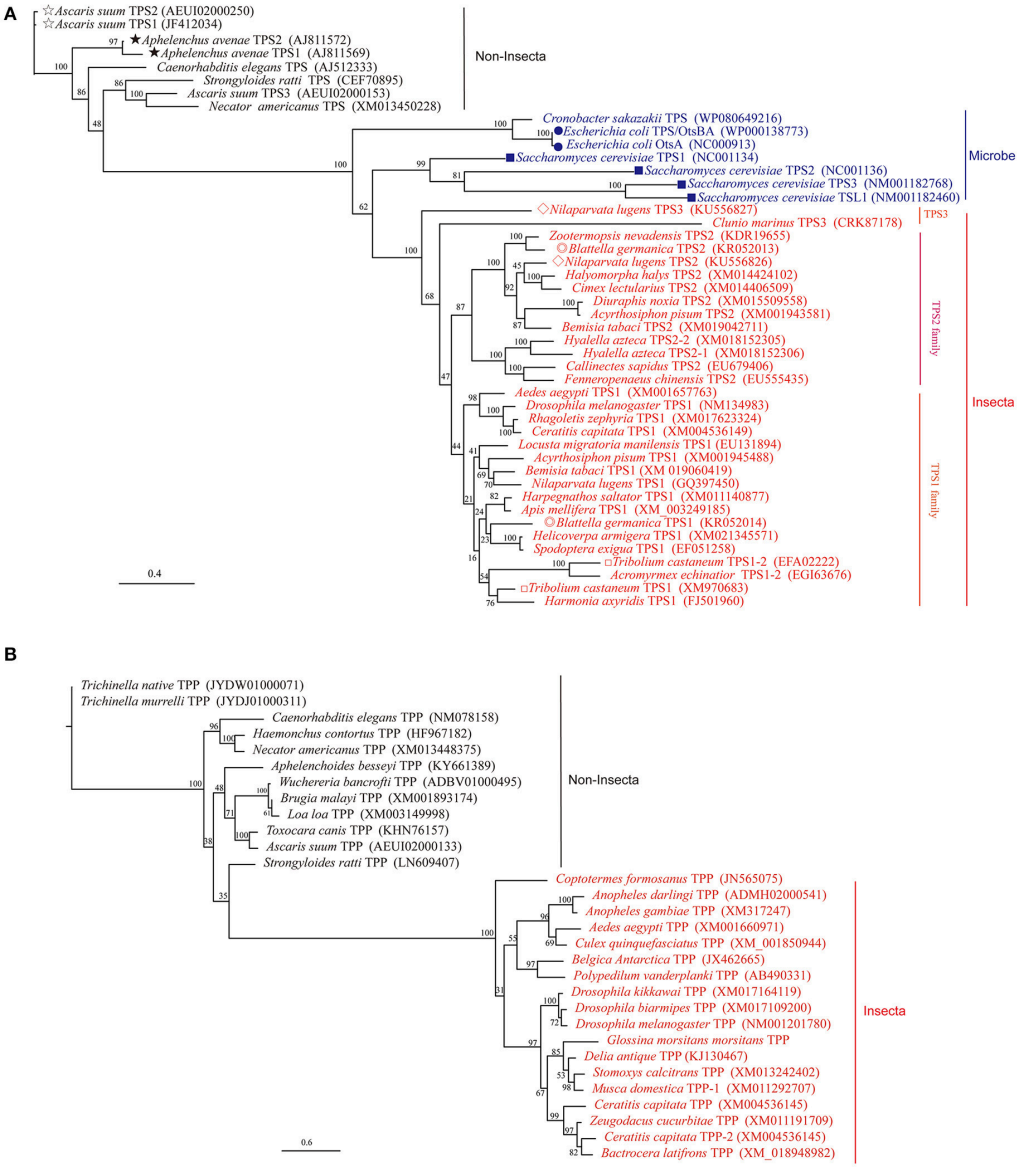
Phylogenetic analysis of insect TPS and TPP based on their amino acid sequences. Full-length amino acid sequences were aligned using Mega 6.0 and to the ML phylogenetic tree (TPS for **A**; TPP for **B**) was performed using PhyML with the model of WAG (Guindon et al., 2005). A bootstrap analysis was carried out and the robustness of each cluster was verified in 1,000 replications. Some species have more than two TPS proteins have marked by different form front of species name **(A)**. The monophyly of insect TPP and TPS is well supported, but the monophyly of non-insect and microbe is not supported.

The first insect *TPS* gene was cloned in *Drosophila* (Chen et al., 2002; Chen and Haddad, 2004), and the induction of *TPS1* gene expression was shown to increase tolerance to anoxia (Chen et al., 2002). Subsequently, insect *TPS* genes were cloned from *Helicoverpa armigera* (Xu et al., 2009), *Locusta migratoria manilensis* (Cui and Xia, 2009), *Spodoptera exigua* (Tang et al., 2010), *Nilaparvata lugens* (Chen et al., 2010b), *Catantops pinguis* (Tang et al., 2011), *Ctenocephalides felis* (Kern et al., 2012), *Harmonia axyridis* (Qin et al., 2012), *Blattella germanica* (Chen and Zhang, 2015), *Delia antiqua* (Guo et al., 2015), *Leptinotarsa decemlineata* (Shi et al., 2016), *Bactrocera minax* (Xiong et al., 2016), and other organisms. Moreover, two *TPS* genes have been found in *B. germanica, Tribolium castaneum*, and *Aphelenchoides besseyi*, and three *TPS* genes have been found in *Ascaris suum* and *N. lugens* (Figure 1A and Table 1, Shen, 2017). In addition, *TPS* from the Chinese shrimp *Fenneropenaeus chinensis* has been cloned and reported (Zhang J. et al., 2012), and one full-length cDNA sequence of four structural isoforms of TPS was isolated from the chela muscles of an adult female (Shi and Chung, 2014). Furthermore, three *TPS* genes have been isolated and sequenced from the muscles of the parasite *A. suum* (Dmitryjuk et al., 2013; Dmitryjuk and Łopienska-Biernat, 2016).

**Table 1:** Characteristics of reported invertebrate TPS gene and their encoding protein.

**Organism**	**TPS Family**	**GeneBank No**.	**No. amino acid**	**References**
*N. lugens*	TPS1	GQ397450	807	Chen et al., 2010a
	TPS2	KU556826	820	Yang et al., 2017
	TPS3	KU556827	783	Shen, 2017
*Sogatella furcifera*	TPS1	JQ743627	807	Zhang D. W. et al., 2012
*Diabolocatantops pinguis*	TPS1	GQ389790	809	Tang et al., 2011
*Locusta migratoria manilensis*	TPS1	EU131894	813	Cui and Xia, 2009
*Antheraea pernyi*	TPS1	KU977454	828	Huang et al., 2016
*Ctenocephalides felis*	TPS1	JX025053	824	Kern et al., 2012
*Delia antique*	TPS1	JX681124	815	Li et al., 2013
*Blattella germanica*	TPS1	KR052013	833	Chen and Zhang, 2015
	TPS2	KR052014	822	Chen and Zhang, 2015
*Leptinotarsa decemlineata*	TPS1	KU756283	821	Shi et al., 2016
*Harmonia axyridis*	TPS1	FJ50196	805	Qin et al., 2012
*Helicoverpa armigera*	TPS1	XM_021345571	826	Xu et al., 2009
*Spodoptera exigua*	TPS1	EF051258	826	Tang et al., 2010
*Drosophila melanogaster*	TPS1	NM_134983	809	Chen et al., 2002, 2003; Chen and Haddad, 2004; Matsuda et al., 2015; Thorat et al., 2016
*Delia antiqua*	TPS1	JX681124	815	Guo et al., 2015
*Ascaris suum*	TPS1	JF412034	1,298	Dmitryjuk et al., 2014
	TPS2	AEUI02000250	1,254	Dmitryjuk et al., 2014
	TPS3	AEUI02000153	1,269	Dmitryjuk et al., 2014
*Callinectes sapidus*	TPS2	EU679406	755	Chung, 2008
*Fenneropenaeus chinensis*	TPS2	EU555435	844	Zhang J. et al., 2012
*Aphelenchoides besseyi*	TPS1	KY661388	1,250	Chen Q. et al., 2017
	TPS2	KY661389	507	Chen Q. et al., 2017
*Caenorhabditis elegans*	TPS1	AJ512333	1,230	Pellerone et al., 2003
*Bactrocera minax*	TPS1	KU379749	814	Xiong et al., 2016

In 2005, gob-1, the first TPP in *Caenorhabditis elegans*, was identified. Loss-of-function mutations in *gob-1* resulted in early larval lethality, which was completely suppressed by the ablation of *C. elegans tps-1* and *tps-2* genes (Kormish and McGhee, 2005). Furthermore, a *TPP* gene was identified in *Brugia malayi* in 2011 (Kushwaha et al., 2011), and its silencing was found to be lethal for the third instar larvae as its *in vivo* development became impaired (Kushwaha et al., 2012). No more *TPP* genes were reported in insects between 2011 and 2015. The identification and cloning of a single *TPP* gene was reported in a diapausing insect, *D. antiqua* (Guo et al., 2015). Furthermore, single *TPP* genes from insects have been reported in GenBank (e.g., *Coptotermes formosanus* [JN565075], *Drosophila melanogaster* [NM_135269], and *Plutella xylostella* [XM_011559193]) (Yang et al., 2017). These genes are shorter than *TPS* and encode proteins containing only the TPP domain, very similar to the TPS protein of the same species, with only some protein sequence differences at the N-terminus (Figure 1B and Table 2).

**Table 2:** Some invertebrate TPP protein sequences from NCBI and published article.

**Organism**	**GeneBank No**.	**No. amino acid**	**References**
*Toxocara canis*	KHN76157	485	
*Aphelenchoides besseyi*	KY661389	507	Chen Q. et al., 2017
*Brugia malayi*	XM_001893174	492	
*Wuchereria bancrofti*	ADBV01000495	467	
*Ascaris suum*	AEUI02000133	532	Dmitryjuk et al., 2014
*Haemonchus contortus*	HF967182	432	
*Strongyloides ratti*	LN609407	451	
*Necator americanus*	XM_013448375	466	
*Delia antiqua*	KJ130467	273	Guo et al., 2015
*Caenorhabditis elegans*	NM078158	468	Kormish and McGhee, 2005
*Loa loa*	XM_003149998	254	
*Trichinella nativa*	JYDW01000071	455	
*Trichinella murrelli*	JYDJ01000311	455	
*Stomoxys calcitrans*	XM_013242402	343	
*Musca domestica*	XM_011292707	331	
*Ceratitis capitata*	XM_004536145	323	
*Zeugodacus cucurbitae*	XM_011191709	333	
*Drosophila kikkawai*	XM_017164119	273	
*Drosophila biarmipes*	XM_017109200	296	
*Drosophila melanogaster*	NM_001201780	296	
*Bactrocera latifrons*	XM_018948982	323	
*Ceratitis capitata*	XM_004536143	273	
*Belgica antarctica*	JX462665	303	
*Polypedilum vanderplanki*	AB490331	294	
*Anopheles darlingi*	ADMH02000541	297	
*Culex quinquefasciatus*	XM_001850944	309	
*Anopheles gambiae*	XM_317247	261	
*Aedes aegypti*	XM_001660971	281	
*Coptotermes formosanus*	JN565075	299	

**Figure 2:**
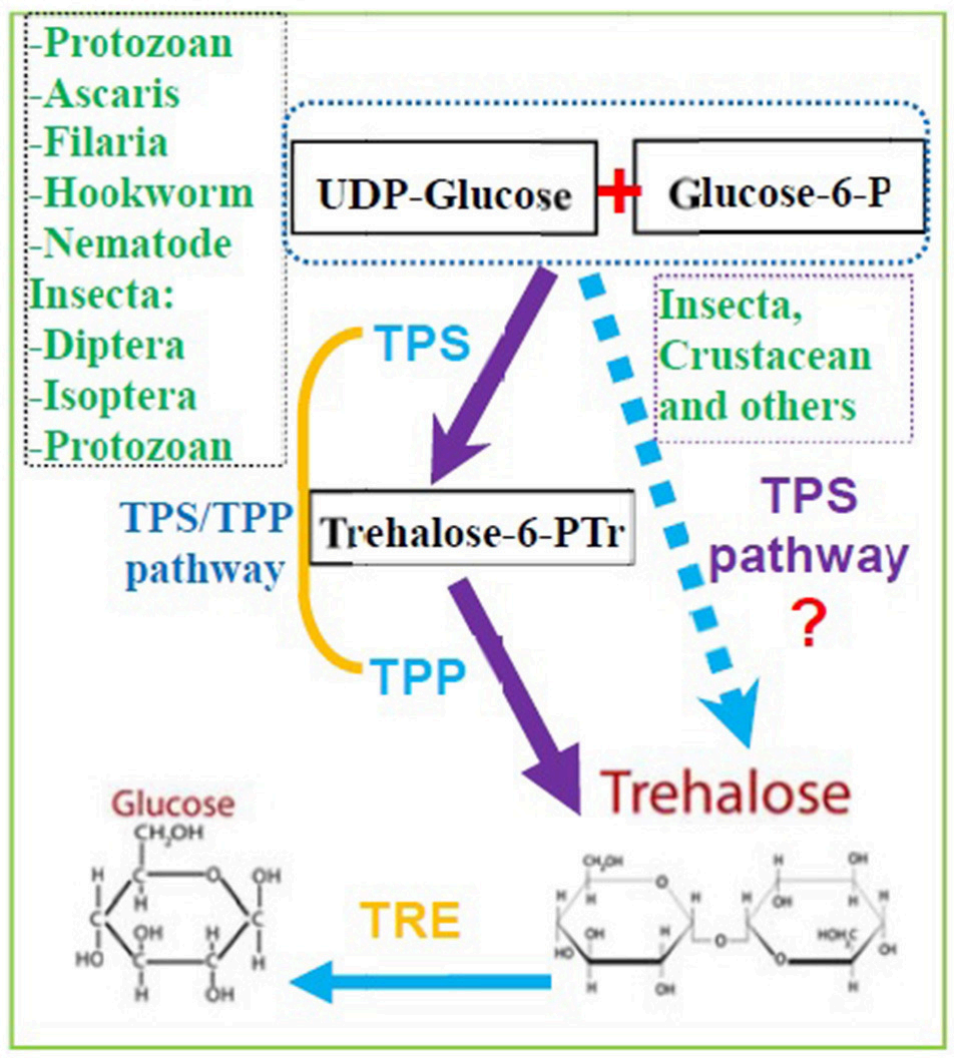
The potential pathway of trehalose synthesis in insects and other invertebrate animals. Trehalose are mainly synthesized by the pathway of TPS and TPP in many kind of invertebrates, but it also be synthesis by TPS only in some species from Insecta and Crustacean. (TPS, Trehalose-6-phosphate synthase; TPP, Trehalose-6-phophate phosphatase; TRE, Trehalase).

Although some invertebrates have more than one *TPS* and *TPP* gene, two *TPS* genes from a single insect were first cloned and reported in *B. germanica* (*BgTPS*1: KR050213 and *BgTPS*2: KR050214) (Chen and Zhang, 2015), followed the discovery of two separate *TPS* genes in *N. lugens* (TPS1: GQ397450, TPS2: KU556826; Yang et al., 2017). The third TPS (KU556827) was cloned from *N. lugens* in 2017 (Shen, 2017). All these *TPS* genes have been found to encode proteins with two conserved TPS and TPP domains with high similarity in their amino acid sequences (Figure 1A, Table 1; Yang et al., 2017). Meanwhile, the use of Illumina RNA-seq technology showed that the beetle *Microdera punctipennis* may have five potential *TPS* UniGenes (Lu et al., 2014). However, the exact number of *TPS* genes in this insect remains unknown. In the evolution of the *TPS* gene, bacteria and yeasts are likely to be closer to the relatives of insects than to nematodes and other non-insects (Figure 1A). Because a) no *TPS* gene has been found in higher animals, like mammals and b) most insects only have one *TPS* gene, which can synthesis trehalose only and has TPS and TPP enzymatic activities (Yoshida et al., 2016). So we hypothesized that the evolution of insect *TPS* evolved from multiple homologs to a single one. Insect or other invertebrate trehalose synthesis from TPS/TPP pathway maybe evolved to one TPS pathway because of *TPS* replaced the function of the *TPP* gene in some species (Figures 1, 2). And this could be the reason for so many insects lacking *TPP* and most of the known *TPP* sequences being closely related to the *TPS* gene sequence. Also it is reported that *Drosophila* have two TPPs (CG5171 and CG5177), but only CG5171 can dephosphoryte T6P under experimental conditions (Yoshida et al., 2016). Of course, more work needs to be done to clearly distinguish the functions of different *TPS* genes in the same species and to elucidate whether all of TPS can synthesize trehalose independently when the species possesses only one *TPS* gene.

### *TPS* gene structure

One study reported that *Drosophila* has only one *TPS* gene, and this gene has domains that are conserved when compared with the yeast genes *TPS* (*OtsA* in *E. coli*) and *TPP* (*OtsB* in *E. coli*) (Chen et al., 2002). Later, *TPS* genes from *H. armigera* (EU878265) and *S. exigua* (EF051258) and many insects have been found and cloned. Insect *TPS* gene encodes an 820–850-aa protein with two conserved domains—TPS and TPP—corresponding to *OtsA* and *OtsB* genes in yeast (Xu et al., 2009; Tang et al., 2010, 2014a; Yang et al., 2017). The *TPS* genes of the blue crab *C. sapidus* were cloned in 2014 and found to be very similar to those of insects. *TPS* genes of four different lengths were isolated: TPS-mus-1 (EU910087), TPS-mus-1a (EU910088), TPS-mus-1b (EU910089), and TPS-mus-1c (EU910090) (Shi and Chung, 2014; Yang et al., 2017). TPS-mus-1b and TPS-mus1 contain conserved TPS and TPP structures, whereas TPS-mus-1b and TPS-mus-1c harbor only a TPS conserved domain (Shi and Chung, 2014; Yang et al., 2017).

**Figure 3:**
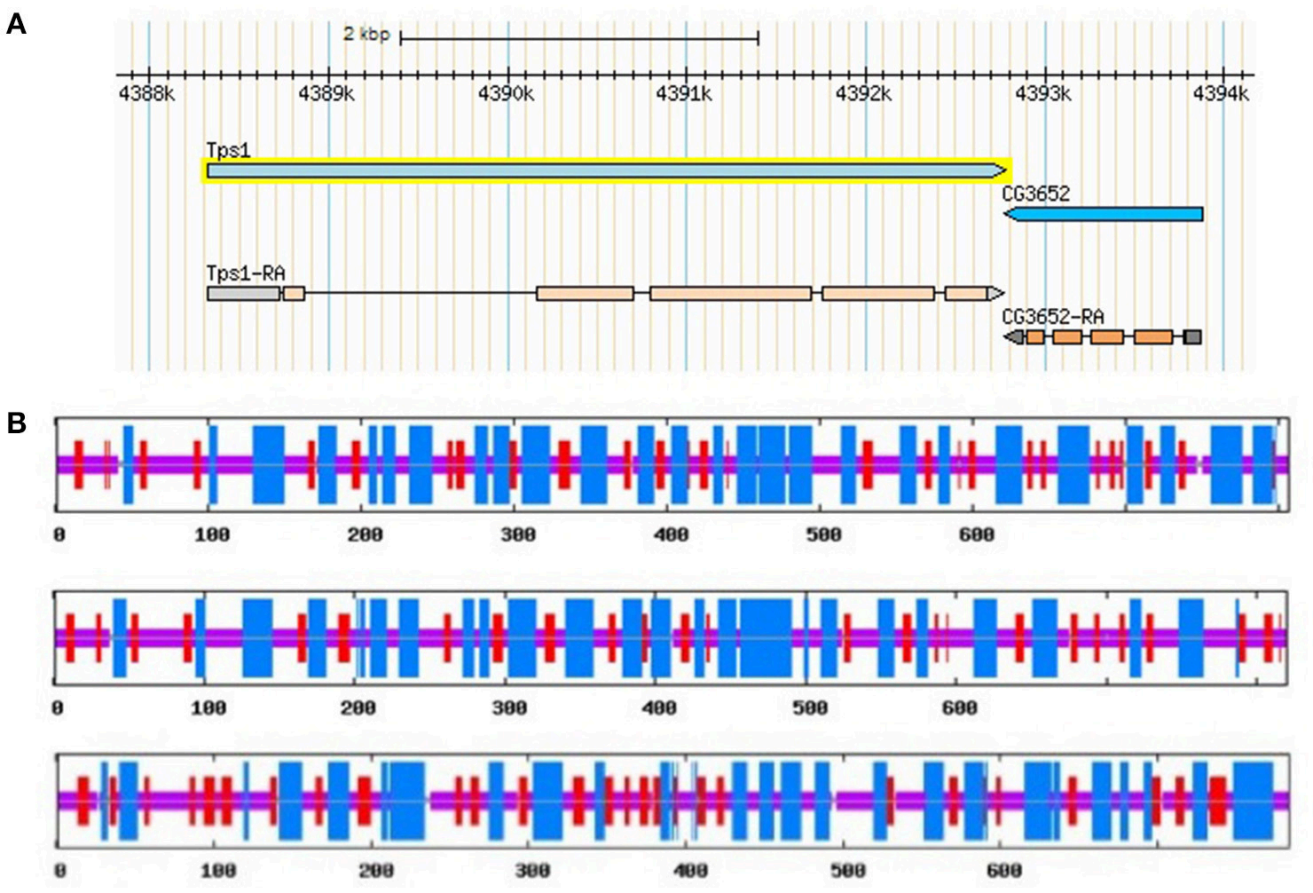
*D. melanogaster* TPS genomic structure **(A)** from FlyBase and predicted secondary structures of three TPS proteins of the brown planthopper, including TPS1, TPS2, and TPS3 **(B)**. Blue and red regions represent α-helixes and β-sheets in Figure 3B, respectively.

The length of TPS genes is variable among different species. It has been shown that *D. melanogaster* TPS (DmTPS or Dmtps1) has 5 exones (Figure 3A). However, *Anopheles gambiae* TPS (AgTPS), *Aedes aegypti* TPS (AaTPS), *Nasonia vitripennis* TPS (NvTPS), *Apis mellifera* TPS (AmTPS) and *S. exigua* TPS (SeTPS) have 5, 5, 3, 10, 8, and 12 exons, respectively. Comparison between SeTPS and NvTPS showed that they have seven common exon–intron boundaries (Tang et al., 2010). The genomic structure of *F. chinensis* TPS (FcTPS) comprises three exons and two introns (Zhang J. et al., 2012). Thus, TPS gene structure has been examined in several insect species, in which genomic sequencing has been completed. Therefore, to determine whether TPS alone can synthesize trehalose, more experiments need to be performed, and structures of gene sequences homologous to TPS and TPP have to be analyzed in insect genomes.

## Biochemistry

### TPS and the trehalose synthesis pathway

Trehalose biosynthetic pathway was first identified in *Saccharomyces cerevisiae* (Cabib and Leloir, 1958; Kern et al., 2012). Initially, insects were thought to synthesize trehalose through the TPS/TPP pathway (Candy and Kilby, 1961), and it was suggested that insects might have the same pathway as that of yeast (Candy and Kilby, 1959, 1961). Now, many studies have reported that there are at least five trehalose biosynthetic pathways in different species (Magalhães et al., 2017). In most invertebrates, including nematodes, TPS/TPP is the predominant trehalose biosynthetic pathway, which involves the following steps (Magalhães et al., 2017): TPS catalyzes the transfer of glucose from UDP-glucose to glucose-6-phosphatase, forming trehalose-6-phosphate (T6P), and TPP dephosphorylates T6P to trehalose (Cabib and Leloir, 1958; Behm, 1997; Elbein et al., 2003; Avonce et al., 2006; Tang et al., 2010; Guo et al., 2015).

The N-terminal TPS (Tre-6-P synthase) domain catalyzes the production of Tre-6-P using glucose 6-phosphate and UDP-glucose as substrates, whereas the C-terminal TPP (Tre-6-P phosphatase) domain then dephosphorylates Tre-6-P, generating trehalose (Matsuda et al., 2015; Yasugi et al., 2017). Expression of *Drosophila TPS* gene in mammalian HEK-293 cells enables them to synthesize trehalose (Matsuda et al., 2015). In *H. armigera*, the level of *TPS* expression corresponds to TPS enzymatic activity as a result of increased trehalose production (Xu et al., 2009). The catalytic activity of *H. armigera* TPS increased approximately fivefold when it was overexpressed in *Bombyx mori* hemolymph infected by using a recombinant baculovirus expression system (Xu et al., 2009). Therefore, we believe that some insects can synthesize trehalose by the TPS/TPP pathway, whereas other insects can synthesize trehalose by TPS alone, just as we described that the evolution of insect TPS evolved from multiple homologs into a single one. In addition, the trehalose synthesis enzyme TPS is solely responsible for the de novo syhthesis of trehalose in *Drosophia* based on the genetic and biochemical evidence (Yoshida et al., 2016). Therefore, a revised trehalose synthesis pathway in insects and other invertebrates is illustrated in Figure 2.

### Tissue expression of *TPS* genes

Because insect fat bodies appear to be important sites for the production of α,α-trehalose, studies of trehalose synthesis have necessarily centered around this organ (Gans et al., 1968), which is analogous to the mammalian liver (Candy and Kilby, 1959; Murphy and Wyatt, 1965; Friedman, 1968). Insects express TPS in the fat body (Cui and Xia, 2009; Xu et al., 2009; Chen et al., 2010b; Tang et al., 2010; Xiong et al., 2016), whereas *C. sapidus* displays ubiquitous expression of TPS in most tissues examined (Chung, 2008). TPS is expressed in all tissues of adult crabs of both sexes, indicating that these tissues can produce trehalose (Chung, 2008). Furthermore, in *L. migratoria manilensis TPS* transcripts are expressed in the fat body, midgut, hemolymph, and leg muscle (Cui and Xia, 2009). It has been reported that *Drosophila* TPP of CG5171 was mainly expressed in the Malpighian tubules and the components of the carcass (Yoshida et al., 2016), so it can't play a role in insect trehalose synthesis because it only works in the fat body.

*F. chinensis TPS* gene was found to be expressed in various tissues, including the muscles, hemocytes, ovaries, gills, nerves, lymphoid organs, intestine, stomach, heart, and epidermis, with the strongest level observed in the hepatopancreas (Zhang J. et al., 2012). Previous reports showed that *L. decemlineata TPS* was highly expressed in the fat body, and it was also transcribed in the foregut, hindgut, trachea, ovaries, and testes, indicating that trehalose might be synthesized in these tissues (Shi et al., 2016). Several reports demonstrated expression of the *TPS* gene in the tissues of foregut and trachea, likely because these two tissues may have been doped with fat body during the extraction process. RT-PCR or northern blot analysis in *C. elegans* showed mRNA expression of two *TPS* genes at all stages of *C. elegans* life cycle (Pellerone et al., 2003; Grewal et al., 2006). *B. minax TPS* expression was detectable in all developmental stages, with a higher expression level in the final (third) instar larvae (Xiong et al., 2016). *In vitro* treatment with a lethal dose of ivermectin decreased TPS and TPP activities in the muscle of adult *A. suum* females compared with those in the control groups (Dmitryjuk et al., 2014).

### Insect development and *TPS* gene expression

In *D. melanogaster*, P element mutagenesis experiments showed that *TPS* gene disruption is lethal at early larval stages (Chen et al., 2002, 2003; Chen and Haddad, 2004). *TPS* mutant *Drosophila* larvae exhibited diet-dependent growth and survival phenotypes when they lacked hemolymph trehalose (Matsuda et al., 2015). Those findings confirmed the assumed crucial functions of TPS synthesis in insects (Becker et al., 1996). In *D. antiqua*, differential expression of TPS and TPP shared similar trends among summer- and winter-diapausing pupae populations, and their enzyme activities were consistent with the expression levels of corresponding genes (Guo et al., 2015). In *C. sapidus*, trehalose concentrations showed a bimodal pattern, and it exhibited two peaks at early ecdysis and post ecdysis, indicating that *C. sapidus* consumes energy from trehalose during the molting process (Chung, 2008). The changes in trehalose content and TPS activity in *H. armigera* hemolymph showed a similar trend during larval-pupal development of diapause and non-diapause programming (Xu et al., 2009).

## Physiological function

### Diversity of *TPS* genes and their functions

TPS plays a key role in the perception of carbohydrate availability and carbohydrate metabolism (Jin et al., 2016) in insects, other invertebrates, as well as in plants (Gao et al., 2014). TPS is considered a cytoplasmic protein with two functionally distinct catalytic domains (Elbein et al., 2003; Matsuda et al., 2015). *Drosophila TPS* gene was cloned and studied at early 2000 (Chen et al., 2002, 2003). Overexpression of *D. melanogaster* TPS in mammalian cells (HEK-293) made them capable of trehalose synthesis (Chen et al., 2003). In *N. lugens*, three *TPS* genes were cloned, and their protein secondary structures showed similar structures and composition of α-helix, β-sheet, and random coil (Table 3, Figure 3B, Shen, 2017). Thus, different *TPS* genes can synthesize trehalose, but it is unclear if different *TPS* genes vary in their function and genomic structure within the same species. Currently, at least three other insect species (*N. lugens, B. germanica*, and *T. castaneum*) have more than two *TPS* genes (Chen and Zhang, 2015; Yang et al., 2017).

**Table 3:** The prediction of the secondary structure of TPS in brown planthopper.

**Item**	**α-helix(%)**	**β-sheet(%)**	**Radom coil(%)**
TPS1	40.27	12.76	45.35
TPS2	36.59	13.29	49.39
TPS3	35.50	18.01	46.49

In addition, TPS1 and TPS2 enzymes have been identified in *C. elegans* (Pellerone et al., 2003), as well as in the anhydrobiotic nematode *Aphelenchus avenae* (Goyal et al., 2005; Kormish and McGhee, 2005). Simultaneous RNA interference (RNAi) targeting of both *TPS1* and *TPS2* in wild-type *C. elegans* lowered trehalose levels to 7% of control levels (Pellerone et al., 2003; Kormish and McGhee, 2005). Nonetheless, on the background of age-1 mutant, RNAi of *TPS1* and *TPS2* greatly decreased *C. elegans* resistance to osmotic shock (Kormish and McGhee, 2005). Meanwhile, there are several instances when multiple *TPS* genes have been found in the same insect species, and these *TPS* genes could have different functions in trehalose synthesis (Chen and Zhang, 2015; Yang et al., 2017). Further research on the distinct roles of different *TPS* genes is warranted.

### Role of TPS in regulating sugar metabolism

It has been reported that larvae lacking trehalose exhibit diet-dependent phenotypes relating to growth and survival in the genetically tractable organism of *Drosophila* (Matsuda et al., 2015). Moreover, a lack of TPS can cause an accumulation of trehalose that is lethal during the pupal period, as well as results in a significant reduction in circulating glucose and the larvae exhibit a high lethality after desiccation stress (Yoshida et al., 2016). Temporary and simultaneous knockdown of both *TPS* genes in *C. elegans* by RNAi resulted in a 90% decline in trehalose levels but no obvious phenotype was observed (Pellerone et al., 2003; Cui and Xia, 2009). In the crustacean *Artemia franciscana*, a fraction of trehalose is quickly mobilized as an energy source, whereas the remainder serves as a substrate for glycogen and glycerol synthesis when dormancy is broken (Collins and Clegg, 2004; Argüelles, 2014). Members of the phylum *Apicomplexa*, a group of protists evolutionarily close to dinoflagellates and ciliates, synthesize trehalose through the biosynthetic pathway similar to that in plants and fungi (Yu et al., 2010; Argüelles, 2014). In nematodes, e.g., in *Anisakis simplex*, glycogen and trehalose metabolism plays a key role in supporting life processes (Łopienska-Biernat et al., 2015). Because TPP has a high affinity for trehalose-6-phosphate and the later hydrolyzes quickly to trehalose, TPS activity is an important limiting factor in trehalose synthesis (Behm, 1997). Two *TPS* genes with very high resemblance to the *tps2* gene of *C. elegans* were also identified in *A. avenae*, but the expression of a gene similar to *C. elegans tps1* has not yet been confirmed (Łopienska-Biernat et al., 2015).

*Drosophila* larvae were shown to be unable to synthesize trehalose when *dTPS1* transcript levels were decreased by the ubiquitous daGAL4-driven expression of the *dTPS1*-RNAi transgene (Thorat et al., 2016). This result highlighted the significance of trehalose in the regulation of desiccation-responsive redox homeostasis (Thorat et al., 2016). The result on the function of TPS further demonstrated that the regulation of trehalose metabolism is essential for normal development, body water homeostasis, and desiccation tolerance in *Drosophila* (Yoshida et al., 2016). Dietary trehalose has also been shown to be directly transported to the hemolymph from the larval gut in insects (Shi et al., 2016), because feeding of trehalose dramatically increased the *in vivo* trehalose pools in *D. melanogaster* larvae treated with DmTPS RNAi (Thorat et al., 2016). In addition, the knockdown of *LdTPS* delayed larval development, strongly reduced hemolymph monosaccharides in the fat body, and potentiated sugar absorption in the larval gut of *L. decemlineata* (Shi et al., 2016). Trehalose can be maintained at a high level while glucose is broken down and used shortly after food intake (Ugrankar et al., 2015; Yasugi et al., 2017). In this condition, the production of trehalose from diet appears to be critical for buffering the fluctuation of sugar levels in the body and for producing trehalose in fat body on a long-term basis (Yasugi et al., 2017). Trehalose is the main hemolymph sugar, and its metabolism plays a pivotal role in systemic energy homeostasis based on the requirement for dietary sugar when both TRE and TPS1 are mutated (Yasugi et al., 2017).

### TPS functions during stress conditions

During 18-h starvation, the maximum distance by which *Harmonia axyridis* moved initially increased and then decreased with time and falling levels of trehalose and glycogen as well as with the reduction in *TPS* expression. This indicates that insects need to consume trehalose to search for food (Tang et al., 2014b; Shi et al., 2017). The Arctic collembolan *Onychiurus arcticus* can survive winter temperatures of −25°C by increasing trehalose concentrations, decreasing glycogen reserves, and reducing TRE activity as temperature decreases. Meanwhile, TPP activity peaks at 0°C (Montiel et al., 1998). TPS induction in *Schizosaccharomyces pombe* transformed with *TPS* gene increased intracellular trehalose levels and the increase correlated with increased tolerance to heat shock and other stresses (Soto et al., 1999). Furthermore, human primary fibroblasts transformed using a recombinant adenovirus vector to express the trehalose biosynthetic enzymes encoded by *OtsA* and *OtsB* genes from *Escherichia coli*., which produced increased amounts of trehalose with increasing multiplicities of infection (Guo et al., 2000). In addition, elevated trehalose levels in mammalian cells transfected with the *Drosophila TPS* gene were reported to protect the cells from hypoxic injury (Chen et al., 2002, 2003; Chen and Haddad, 2004).

In *Polypedilum vanderplanki*, one of the mechanisms of the tolerance to extreme conditions is that the larvae can rapidly accumulate trehalose to the levels up to 18% of dry body mass (Watanabe et al., 2002; Chen and Haddad, 2004). In 2009, Xu et al. reported TPS activity regulates the changes in trehalose content during *H. armigera* larval-pupal development, and that this is the reason of a significantly higher trehalose concentration in diapausing insects than in non-diapausing insects (Xu et al., 2009). Furthermore, it has been reported that trehalose concentrations were lower in summer- and winter-diapausing pupa at the initial phase, but then, they increased gradually and peaked during the maintenance phase (Guo et al., 2015). The concentration then declined in the quiescence phase, indicating that trehalose metabolism plays an important role through the expression of *TPS, TPP*, and *TRE* genes (Guo et al., 2015). In overwintering mountain pine beetle larvae, TPS levels are high in the autumn proteome, whereas in the spring proteome, they are significantly lower. This observation supports the hypothesis that trehalose is produced for survival during cold periods (Bonnett et al., 2012). TPS has also been found to possess anti-stress functions and play putative roles in physiological adaptation to environmental stress in *Bactrocera dorsalis* (Yang et al., 2014) and *C. sapidus* (Yednock and Neigel, 2014).

*Anastrepha ludens* larvae developed a protection mechanism based on the synthesis of trehalose by TPS to achieve greater survivability to stress caused by hydrostatic pressure (Vargas-Ortiz et al., 2013). Starvation and the injection of dsSeHTF—an Akh-like hypertrehalosemic factor—can significantly decrease *TPS* expression level (Park and Kim, 2017). Although no conspicuous phenotype changes were observed after *TRE* and *TPS* genes were silenced individually or simultaneously in the nematode *A. besseyi*, its survival under hypertonic osmotic pressure decreased significantly and the recovery was delayed. Thus, trehalose metabolism genes, including *TPS* and *TRE*, play an important role in osmobiosis regulation in a time/season-dependent fashion (Chen Q. et al., 2017).

### Regulation of chitin metabolism by TPS

**Figure 4:**
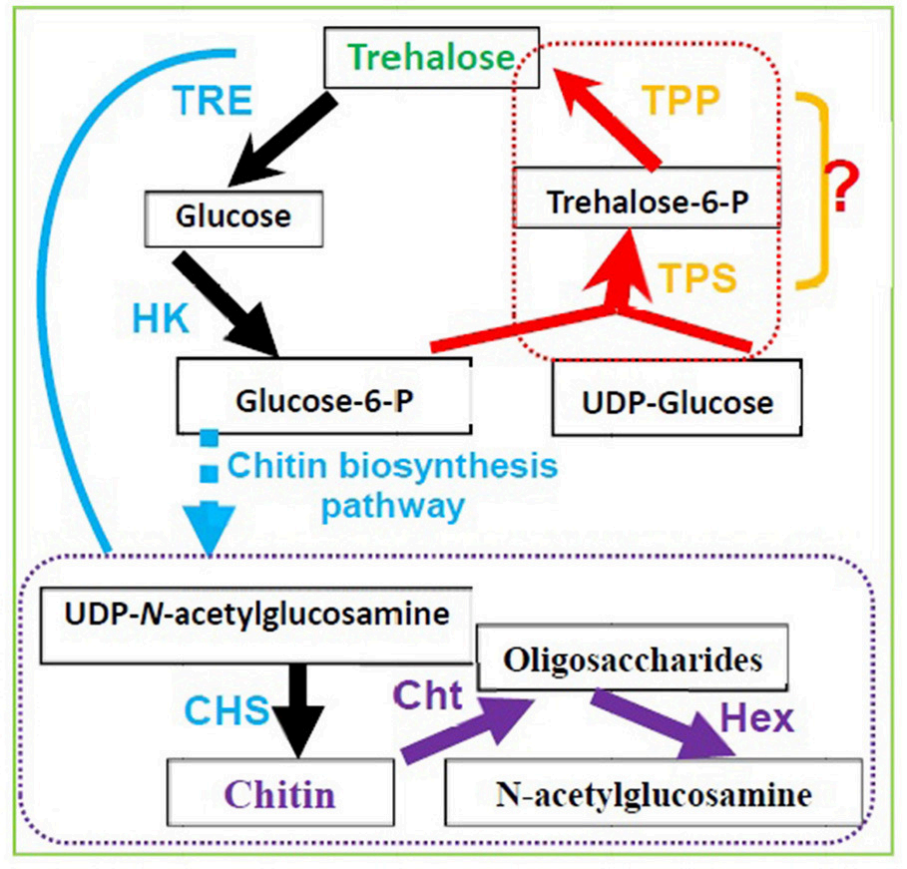
Trehalose metabolism and its relevance to chitin biosynthesis and degradation in insects and invertebrate animals. (TRE, Trehalase; HK, Hexokinase; CHS, Chitin synthase; Cht, Chitinase; Hex, β-N-acetylhexosamindase or β-N-acetyl-D-Hexosamindase; TPS, Trehalose-6-phosphate synthase; TPP, Trehalose-6-phophate phosphatase).

In silkworm larvae, trehalose has been reported to be a source of carbon for chitin synthesis during the new cuticle production and molting stages (Duchateau-Bosson et al., 1963). Trehalose is also considered a major substrate for chitin synthesis (Shi et al., 2016; Xiong et al., 2016). In insects, ecdysis, i.e., shedding of the cuticle at the end of a larval stadium, only occurs when ecdysteroid returns to a low level after its peak titer in the hemolymph (Steele, 2016). In *Periplaneta americana*, ecdysis is strongly correlated with the increase in trehalose and glucose concentrations in the hemolymph (Steele, 2016), suggesting a causal relationship between both events. 20-Hydroxyecdysone has been shown to induce the expression of *BmTPS* and three other genes in the chitin biosynthesis pathway, including *TRE*, glucose-6-phosphate isomerase (*G6PI*), and chitin synthase (*CHS*) (Xiong et al., 2016). *TRE* is the first gene in the chitin synthesis pathway (Tang et al., 2008, Zhang et al., 2011), and it regulates insect chitin synthesis and degradation (Tang et al., 2016; Zhao et al., 2016). Figure 4 illustrates how TPS in the chitin synthesis pathway regulates insect molting (Chen Q. W. et al., 2017; Yang et al., 2017).

*S. exigua* complete the process of molting and die when *TPS* is knocked down by RNAi (Tang et al., 2010): the decrease in trehalose content causes larval and pupal lethality. In *L. decemlineata*, when *LdTPS* was knocked down by RNAi, surviving insects consumed a greater amount of foliage; accumulated more glycogen, lipid, and proline; and gained a larger body mass with a lower amount of chitin than did control insects (Shi et al., 2016). Moreover, TPS activity and trehalose content decreased significantly when dsRNA was injected into third -instar larvae, successfully silencing the transcription of BmTPS in *B. minax* and inhibiting the expression of three key genes in the chitin biosynthesis pathway. Furthermore, this treatment was associated with 52% mortality rate and the appearance of abnormal phenotypes (Xiong et al., 2016). In *N. lugens*, three phenotypes, namely molting deformity, molting and wing deformity, and wing deformity, occurred when the expression of *TPS1* or *TPS2* was decreased significantly by RNAi, along with 30% mortality (Chen et al., 2010b; Yang et al., 2017) and a significant decrease in trehalose content (Zhang et al., 2017). In addition, the expression of chitinase genes and chitin content decreased significantly, after that mainipulation, suggesting that the chitin metabolism balance is disrupted upon *TPS* gene knockdown (Chen Q. W. et al., 2017; Shen, 2017; Yang et al., 2017).

An increasing number of key enzymes and proteins of crop insects are being identified as candidates for RNAi-based gene silencing (Kola et al., 2015; Joga et al., 2016; Reisenman et al., 2016; Kolliopoulou et al., 2017). In a study by Shi et al. (2016), in *TPS* RNAi group, the chitin content in the body and epidermis decreased significantly, compared with that in the control group, from the third day to the eighth day of the life cycle. Moreover, a rescue bioassay revealed that trehalose feeding increased the survival of TPS RNAi hypomorphs and partially recovered chitin content.

## Potential target for insecticides

The non-reducing disaccharide trehalose is absent in vertebrates, and, in particular, in mammals (Argüelles, 2014). This physiological difference might provide clues regarding the evolutionary branching of invertebrates and vertebrates (Argüelles, 2014). An increasing number of studies have shown significant mortality in insects when the trehalose balance is blocked (Chen et al., 2010a; Tang et al., 2010), further supporting the notion that TPS enzyme inhibition might be a viable insecticidal mechanism (Kern et al., 2012). However, until recently, no attempts to use inhibitors of insect TPS have been undertaken. In 2012, 4-substituted 2,6-diamino-3,5-dicyano-4H-thiopyrans were applied at potential inhibitory concentrations on insect TPS and highlighted as potential lead compounds for the development of insecticides (Kern et al., 2012). TPP is suggested to be a promising target for the development of antibacterial, antifungal, and antihelmintic therapeutics (Liu et al., 2017). The World Health Organization has included *B. malayi* TPP enzyme in the priority list of prospective antifilarial drug targets for lymphatic filariasis (Ho et al., 1992).

Studies on some potent inhibitors of insect TREs such as trehazolin (Ando et al., 1991, 1995), validoxyamine-A (Asano et al., 1990), and its derivative validamycin, have suggested that these compounds can act as insecticides by interfering with trehalose utilization in flight muscles, wing buds, cuticle, nervous system, and other body parts (Kono et al., 1999; Wegener et al., 2003, 2010; Tang et al., 2017). Application of *TPS* RNAi constructs via injection into *S. exigua* larvae (Tang et al., 2010) or via feeding into *N. lugens* larvae (Chen et al., 2010b) led to significant mortality in these insect species, further supporting the notion that TPS enzyme inhibition might be a viable insecticidal mechanism (Kern et al., 2012). Aryl d-glucopyranoside 6-sulfate prototypes are expected to find future applications for the development of tailored second-generation T6PP inhibitors (Liu et al., 2017). Interfering with trehalose biosynthesis could also be an insecticidal approach, making the trehalose biosynthesis enzyme TPS a potential drug target for pest control (Kern et al., 2012). A considerable body research over the recent years has demonstrated that TPS is indispensable for larval-pupal metamorphosis and that it is a suitable target to control insect and helminth pests by inhibiting the trehalose synthesis pathway (Tang et al., 2010; Xiong et al., 2016; Yang et al., 2017).

To conclude, *TPS* genes have been identified so far in hundreds of insect and other invertebrate species. We have reviewed the current understanding of the evolutionary and physiological significance of trehalose. In future studies, different trehalose synthesis pathways, distinct functions of multiple *TPS* genes, and sensitivity of TPS proteins to potential pest control inhibitors should be investigated in depth.

## Author contributions

Conceived and manuscript structure design: SW, S-GW, S-YC, and BT. Current articles collection and trehalose metabolism genes' analysis: SW, H-JW, and BT. Sequence data analysis and figure drawing: BT and J-YZ. Wrote the paper: S-YC and BT.

### Conflict of interest statement

The authors declare that the research was conducted in the absence of any commercial or financial relationships that could be construed as a potential conflict of interest.
